# Management of acute alcohol withdrawal in the setting of a quick diagnostic unit integrated in an emergency department setting

**DOI:** 10.1186/1757-7241-21-S2-A6

**Published:** 2013-09-09

**Authors:** Pernille Würtz Bøhm, Tove Beyer Fuglevig Mortensen, Thomas Andersen Schmidt

**Affiliations:** 1 The Emergency Department, Holbæk Hospital, Denmark

## Background

Alcohol consumption can have adverse social, legal, occupational, psychological, and medical consequences. The prevalence of alcohol-use disorders is high in Emergency Departments. The novel establishment of a Quick Diagnostic Unit (QDU) in an ED setting has allowed expeditious and focused, medically supervised acute alcohol withdrawal. The purpose of the study was to describe the alcohol-use disorder clientele and treatment in this new setting.

## Methods

Chart review of an 8 month period April to December 2012. Values were given as means ± SEM. Significance was evaluated using Student’s two-tailed t-test for unpaired observations or Fisher’s exact test as appropriate. The level of significance was established at p < 0.05.

## Results

A total of 91 patients were included in the study, 74 men and 17 women. The patients in total amounted to 2.6% of the discharged patients from the QDU. There was no age difference between men and women, i.e. 51.2 ± 1.5 years vs 50.6 ± 2.5 years (p>0.80). Length of stay was 1.8 ± 0.2 days for men vs 2.5 ± 0.6 days for women (p>0.2). In 19% of the cases men held jobs, whereas none of the women were employed (p < 0.0001).

Among patients who received chlordiazepoxide (RisolidR) for withdrawal symptoms the total dose was 405 ± 43 mg (n = 53) among men vs 494 ± 105 mg (n = 14) among women (p > 0.30). Thus 72% of the men vs 82% (p > 0.10) of the women were in need of chlordiazepoxide. There were no differences between men and women with regard to need for ICU care or emergent psychiatric referral (p > 0.20). Men left the QDU against medical advice to a greater extent than women, i.e. 22% vs 6% (p < 0.002).

## Conclusion

Women with alcohol-use disorders appear to be more marginalized than men. Thus, they are employed to a lesser extent than men, and numerically they are treated more frequently and with a higher total dose of chlordiazepoxide. Men are more capable or prone than women to reject treatment. Further studies of the QDU setting would be beneficial.

